# Gene expression profiling of cutaneous wound healing

**DOI:** 10.1186/1479-5876-5-11

**Published:** 2007-02-21

**Authors:** Kavita Deonarine, Monica C Panelli, Mitchell E Stashower, Ping Jin, Kina Smith, Herbert B Slade, Christopher Norwood, Ena Wang, Francesco M Marincola, David F Stroncek

**Affiliations:** 1 Immunogenetics Section, Department of Transfusion Medicine, Clinical Center National Institutes of Health, Bethesda MD, 20892, USA; 2 The Clinical Skin Center of Northern Virginia, Fairfax, VA, 22033, USA; 3 DFB Pharmaceuticals, Fort Worth, TX, 76107, USA

## Abstract

**Background:**

Although the sequence of events leading to wound repair has been described at the cellular and, to a limited extent, at the protein level this process has yet to be fully elucidated. Genome wide transcriptional analysis tools promise to further define the global picture of this complex progression of events.

**Study Design:**

This study was part of a placebo-controlled double-blind clinical trial in which basal cell carcinomas were treated topically with an immunomodifier – toll-like receptor 7 agonist: imiquimod. The fourteen patients with basal cell carcinoma in the placebo arm of the trial received placebo treatment consisting solely of vehicle cream. A skin punch biopsy was obtained immediately before treatment and at the end of the placebo treatment (after 2, 4 or 8 days). 17.5K cDNA microarrays were utilized to profile the biopsy material.

**Results:**

Four gene signatures whose expression changed relative to baseline (before wound induction by the pre-treatment biopsy) were identified. The largest group was comprised predominantly of inflammatory genes whose expression was increased throughout the study. Two additional signatures were observed which included preferentially pro-inflammatory genes in the early post-treatment biopsies (2 days after pre-treatment biopsies) and repair and angiogenesis genes in the later (4 to 8 days) biopsies. The fourth and smallest set of genes was down-regulated throughout the study. Early in wound healing the expression of markers of both M1 and M2 macrophages were increased, but later M2 markers predominated.

**Conclusion:**

The initial response to a cutaneous wound induces powerful transcriptional activation of pro-inflammatory stimuli which may alert the host defense. Subsequently and in the absence of infection, inflammation subsides and it is replaced by angiogenesis and remodeling. Understanding this transition which may be driven by a change from a mixed macrophage population to predominately M2 macrophages, may help the interpretation of the cellular and molecular events occurring in the microenvironment of serially biopsied tissues.

## Background

In the classical model of wound healing four overlapping phases are described: hemostasis, inflammation, proliferation, and remodeling [1]. Cutaneous wounds are associated with a series of cellular responses that include blood clotting, platelet activation, inflammatory cell infiltration, re-epithelization and the formation of granulation tissue made up of fibroblasts and blood vessels. The later stages of healing include matrix deposition and wound contraction and re-epithelialization [1-4].

In skin, wound healing begins with hemostasis marked by the appearance of platelets [1]. A platelet aggregate is formed at the site of the wound and activated platelets release cytokines involved with wound healing such as platelet-derived growth factor (PDGF) and transforming growth factor beta (TGF-β). At the site of the platelet clot, coagulation system enzymes are activated and fibrinogen is converted to fibrin. The resulting network forms the provisional matrix for tissue repair [5]. Several hours after clot formation, keratinocytes begin to move into the site from the edges of the injury to begin to close the wound [6]

The inflammatory response includes neutrophils, macrophages, and mast cells. Neutrophils are first to infiltrate the site of the wound. Within 24 hours neutrophils become the predominant leukocyte in the wound where they remove foreign material, bacteria, and damaged matrix [1]. Tissue macrophages arrive within 48 hours and produce both cytokines and growth factors. They also play a role in deébridement, acting as phagocytes to clear away matrix deébris. The appearance of activated macrophages is accompanied by the appearance of lymphocytes and marks the end of the inflammatory phase and the beginning of the proliferative phase of wound healing.

The proliferative phase is associated with the production of collagen, proteoglycans and fibronectin to form new extracellular matrix, continue epithelization, and angiogenesis. Fibroblasts, which produce matrix and collagen, are the predominant cell in this phase. TGB-β which is produced by platelets, macrophages, and T cells is a potent stimulus of fibroblasts and plays a critical role in the proliferative phase [3]. Angiogenesis begins with the migration of endothelial cells into the fibrin matrix. Endothelial cells begin to degrade the interstitial matrix in order to form new capillaries. TGF-β along with vascular endothelial growth factor (VEGF) and basic fibroblast growth factor stimulate angiogenesis [3].

The final phase of wound healing, remodeling, involves the degradation of collagen by proteolytic enzymes produced by fibroblasts, neutrophils and macrophages. This phase is also characterized by the infiltration of mast cells which manage host wound repair through increased inflammatory signaling [1].

Although wound healing is a complex process involving hemostasis, inflammation, cell proliferation, cell migration, and tissue remodeling, most studies of wound healing have focused on specific cells or specific proteins or genes. In this study of wound healing the expression of a broad spectrum of genes was assessed before and over 2, 4 and 8 days after a skin wound. Samples were obtained as part of a placebo-controlled double-blind study of gene expression profiling of basal cell carcinoma treated topically with Imiquimod, a drug whose activity is mediated through toll-like receptor 7 (TLR-7) signaling [7]. We studied fourteen patients with basal cell carcinoma in the placebo group who were treated topically with control cream using treatment schedules of 2, 4 or 8 days. In each of the fourteen patients studied, a skin punch biopsy was obtained immediately before treatment was started and at the end of the placebo treatment period. Gene expression cDNA microarrays were used to analyze the skin punch biopsies of tissue taken from the basal cell carcinoma of each patient. Since the skin biopsies were taken immediately adjacent to each other, the initial skin biopsies were considered to be pre-wound and the second biopsy was considered to represent the host response to the wound.

## Materials and methods

### Study design

Skin biopsies were obtained from 14 subjects belonging to the control group of a double-blind clinical trial in which basal cell carcinomas were treated topically with the immunomodifier-TLR-7 agonist: imiquimod [7]. The basal cell carcinoma was located on the proximal extremities, trunk, scalp or face and was at least 7 mm in diameter. Placebo cream covering at least 2 cm was applied to the basal cell carcinoma for either 2, 4, or 8 days (Table 1). Punch biopsies (2 mm) of the basal cell carcinomas were performed at the beginning of the study and approximately 12 hours after the final application of placebo (Table 1). The first (pre-wound) biopsy was considered to create the wound and the second biopsy, which was subsequently taken immediately adjacent to the first biopsy, was considered the host response to that wound (post-wound). Among the 14 patients, three were biopsied on day 2, seven on day 4, and four on day 8. Gene expression profiling was performed on the pre- and post-treatment punch biopsies obtained from the 14 control patients. RNA was amplified and hybridized to 17.5K cDNA arrays.

**Table 1:** Duration and Schedule of Topical Placebo Application

**Patient**	**Placebo Application Schedule**	**Doses of Placebo Cream**
P10	Every 12 hr × 2 days	4
P23	Every 12 hr × 2 days	2
P26	Every 12 hr × 2 days	4
P41	Every 12 hr × 4 days	8
P134	Every 12 hr × 4 days	8
P8	Every 12 hr × 4 days	8
P20	Every 12 hr × 4 days	8
P4	Every 24 hr × 4 days	4
P13	Every 24 hr × 4 days	4
P36	Every 24 hr × 4 days	4
P2	Every 24 hr × 8 days	8
P15	Every 24 hr × 8 days	8
P27	Every 24 hr × 8 days	8
P137	Every 24 hr × 8 days	8

### RNA isolation and amplification and cDNA microarray analysis

Total RNA was isolated with RNeasy minikits (Qiagen, Germantown, MD) and amplified into anti-sense RNA as previously described [8,9] with the following modifications to minimize RNA degradation by abundant skin RNAases. Samples were homogenized in disposable tissue grinders (Fisher Scientific, Lafayette, CO, USA). Proteins potentially interfering with RNA isolation were removed by incubating the homogenate in 590 μl distilled water and 10 μl PROTEINASE K solution (Qiagen) at 55°C for 10 minutes then centrifuged at ambient temperature for 3 minutes. Supernatants were combined with 0.5 volumes of ethanol (96–100%) into a Rnase-Dnase free tube and RNA was isolated through a RNeasy mini column. First strand cDNA synthesis was accomplished in 1 μl SUPERaseIn (Ambion, Austin, Tx, USA) and ThermoScript RT (Gibco BRL, Gaithersburg, MD, USA) in 2 μg bovine serum albumin. RNA quality was verified by Agilent technologies (Palo Alto, CA). Anti-sense RNA was used for probe preparation Test samples were labeled with Cy5-dUTP (Amersham, Piscataway, NJ, USA) and co-hybridized with reference pooled normal donor peripheral blood mononuclear cells (PBMC) labeled with Cy3-dUTP to custom made 7K-cDNA microarrays [10]. Arrays were scanned on a GenePix 4000 (Axon Instruments, Union City, CA) and analyzed using Cluster and Tree View software [11]. Gene ratios are presented according to the central method for display [12]. Gene annotations were mined using the following web-based tools [13-16].

### Statistical analysis

Significance testing was based on paired or 2-sample two-tailed Student t tests as appropriate. P-values < 0.05 were considered statistically significant. Microarray raw data was curated according to GEO [17].

## Results

### Gene expression profiles in skin before and after a wound

Pre- and post-wound gene expression profiles were compared in skin biopsies from 14 subjects with basal cell carcinoma using unsupervised hierarchical clustering. Eisen's correlation and clustering was applied to the 17.5K global data set using various filtering parameters, however, independent of the filter applied, no correlation or molecular signatures that separated pre- and post-wound samples was identified. Pre- and post-wound samples from 4 of the 14 subjects clustered as pairs indicating that in some cases differences between subjects were greater than differences within a subject due to wound healing (data not shown).

**Figure 1:**
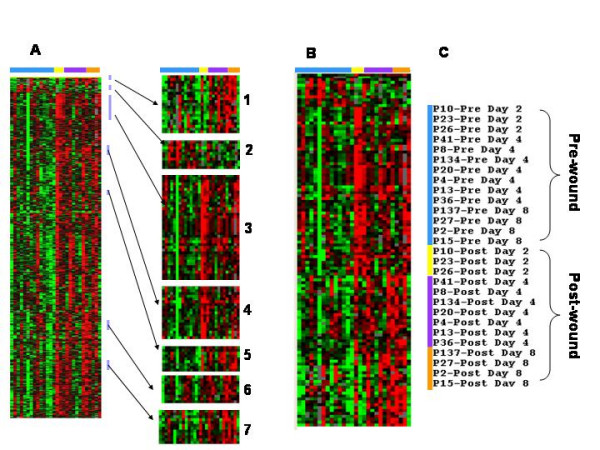
**Genes differentially expressed between pre and post wound skin biopsies**. One skin punch biopsy was obtained pre wound and one either day 2, 4, or 8 post wound from 14 subjects. The 1,749 genes found to be differentially expressed using paired t-test comparison (p < 0.05) were analyzed by filtering (genes expressed in 80% of samples and at least more increased 2 or more fold) and this yielded 753 genes. Supervised hierarchical clustering analysis of Eisen of the 753 differentially expressed genes revealed 7 signature clusters (Figure 1a). The genes in the 7 clusters were combined and analyzed again by supervised hierarchical clustering (Figure 1b). The pre wound biopsies are indicated by the blue bar, the day 2 post wound biopsies by the yellow bar, the day 4 post wound biopsy by the purple bar, and the day 8 post wound biopsy by the orange bar (Figure 1c).

Gene expression profiles were then analyzed using supervised clustering on the global data set and by Student t tests (cutoff p-value < 0.05 between genes expressed in pooled pre-versus post-wound samples). Supervised Eisen's correlation and clustering revealed that the expression of most of the genes was either increased or unchanged in the post-wound samples. Comparison by paired Student t tests identified 1,749 genes differentially expressed between pre- and post-wound samples. Filtering of this gene set identified 753 transcripts that were expressed in 80% of the samples and were differentially expressed between pre- and post-treatment biopsy at least 2 or more fold. Visual inspection identified 7 prominent signatures containing a total of 120 genes (Figure 1).

**Figure 2:**
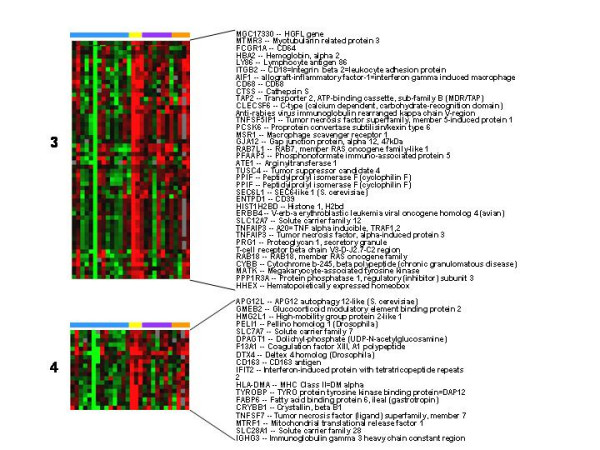
**Signature clusters of genes whose expression was changed early in wound healing**. Genes in signature clusters 3 and 4 whose expression was greatest on day 2 are shown. Pre and post wound biopsies are color coded by the horizontal bars as per figure 1.

Two clusters included predominantly transcripts who expression was increased early on day 2 after wounding (Figure 2, clusters 3 and 4) and represented a wide variety of pro-inflammatory genes. Several markers were present indicating TNF activation (TNF receptor associated factor 1,2) and interferon (IFN) activation, (AIF1 and IFIT2). The expression of several macrophage markers was increased (CD163, FcγRI, macrophage scavenger receptor 1, and MHC class II α chain) as were markers of T cells (RAB7L1, and RAB18), B cells (immunoglobulin heavy chain) and neutrophils (cytochrome b-245). In addition, the expression of β_2 _integrin, CD18, which is found on many different leukocytes, was increased. Evidence of tissue repair was also present including the presence of the cysteine protease, cathepsin S, which is produced by smooth muscle cells and induces angiogenesis [18] and coagulation factor XIII, a platelet and monocyte protein that is involved with fibrin crosslinking and clot maturation and that increases endothelial cell migration and proliferation [19].

**Figure 3:**
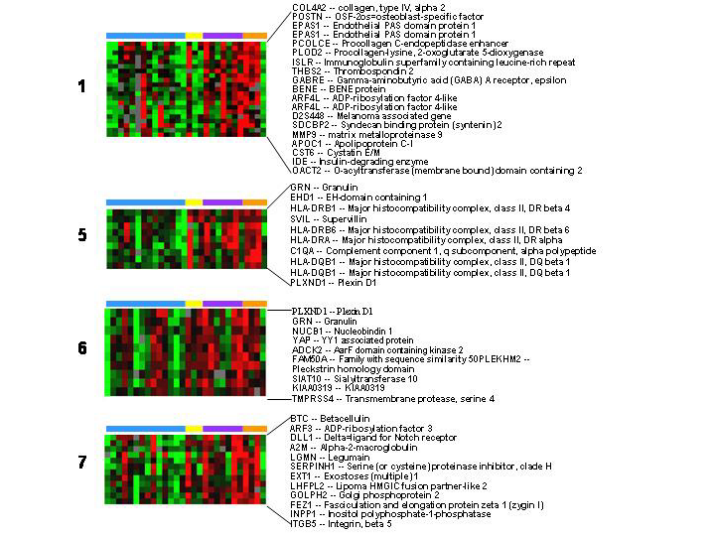
**Signature clusters of genes whose expression was changed late in wound healing**. Genes in signature clusters 1, 5, 6, and 7 whose expression was greatest on days 4 and 8 are shown. Pre and post wound biopsies are color coded by the horizontal bars as per figure 1.

The expression of genes in 4 clusters was increased most on days 4 and 8 post-wound (Figure 3, clusters 1, 5, 6, and 7). These "late" wound healing genes included markers of tissue repair, angiogenesis, and further progression of immune response. Tissue repair/remodeling genes whose expression was increased included type IV collagen and collagen modifying enzymes (procollagen C-endopeptidase enhancer and procollagen-lysine, 2-oxoglutarate 5-dioxygenase) and the integrin β_5_. Angiogenesis genes whose expression was increased included matrix metalloprotease 9 (MMP-9) and granulin or progranulin. Progranulin is a pluripotent growth factor that promotes cell proliferation, cell motility, granulation and angiogenesis [4]. Immune response genes among the late wound healing genes included several MHC class II molecules and complement component 1q.

**Figure 4:**
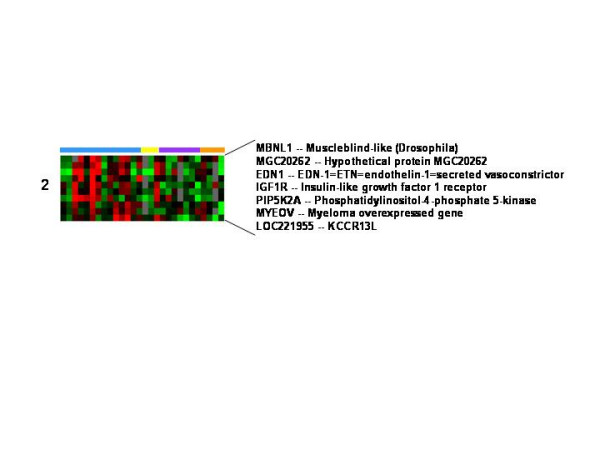
**Genes whose post wound expression was decreased on all days**. The expression of genes in signature cluster 2 were decreased on all days. Pre and post wound biopsies are color coded by the horizontal bars as per figure 1.

The expression of genes in signature cluster 2 were reduced in all post-wound samples (Figure 4). The down-regulated cluster included endothelian-1 which is a vasoconstrictor suggesting that vasodilation and increased blood flow is an important aspect of wound healing.

**Figure 5:**
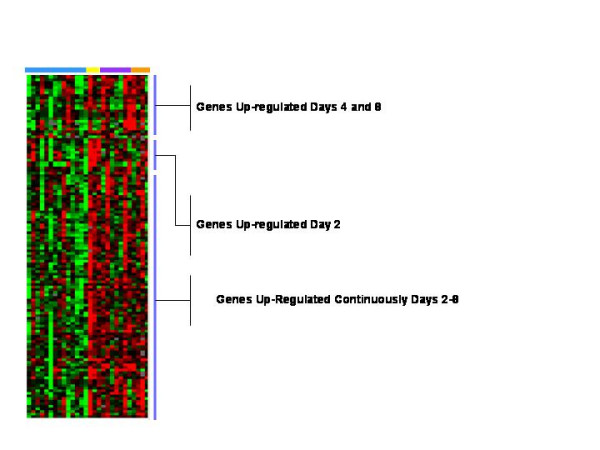
**Supervised Eisen hierarchical clustering analysis of 133 differentially expressed genes between pre and post wound that were not included in the signature clusters**. From the 753 differentially expressed genes, the genes in the signature clusters were removed and genes important in inflammation, immune response and wound healing were selected according to DAVID [13] and Gene Cards [14] and analyzed by supervised hierarchical clustering. Three clusters were identified; one whose expression was increased most on day 2, one on days 4 and 8, and another whose expression was increased on day 2–8. The specific genes are shown in additional file 2. Pre and post wound biopsies are color coded by the horizontal bars as per figure 1.

The 753 genes included, in addition to the 7 time-dependent signatures, another 131 genes involved in tissue repair/remodeling, proliferation, inflammation, or immune response according to DAVID [13] and Gene Cards [14] mining tools. Supervised hierarchical clustering of the 131 transcripts emphasized the differential kinetics of their appearance. Genes were thus segregated into three groups: early transcripts, late transcripts and transcript whose expression remained sustained independently of time (Figure 5 and see additional file 1).

The early group of genes whose expression was increased most on day 2 included several leukocyte markers; the leukocyte adhesion molecules CD11C, CD44, and CXCR4 and the macrophage marker CD68. In addition, this group included type 1 TGF-β.

The late group of genes whose expression was increased most on days 4 and 8 included several leukocyte genes, IL-10 receptor β chain, and MMP-2, and genes associated with tissue repair, type 5 collagen, procollagen, connective tissue growth factor, and integrin αv. The expression of the endothelial cell tight junction protein CD99 was also increased and the tissue repair proteins ADAMTS3 and ADAM15.

The largest cluster was made up of genes whose expression was increased at all time points in post-wound samples. Most of these genes were associated with inflammation including several proteasomes, interferon-inducible proteins, mitogen-activated protein kinases, and HLA class I and II molecules as well as CD6, MMP-11, macrophage migration inhibitory factor (MIF), PECAM-1, lactoferrin, and toll-like receptor 1. In addition, the expression of capsases, death effector domain, tissue inhibitor of metalloprotease -1 (TIMP1), and TIMP2 were also increased. This group included markers of repair including collagen, urokinase type plasminogen activator and hypoxia-inducible factor (HIF). HIF is a nuclear transcription factor whose expression is increased in endothelial cells and that stimulates the expression of VEGF and hence angiogenesis [1]. In addition, the expression of HSPA1B (HSP70) which accelerates wound healing was also increased [20].

### Comparison of markers of M1 and M2 macrophages

**Figure 6:**
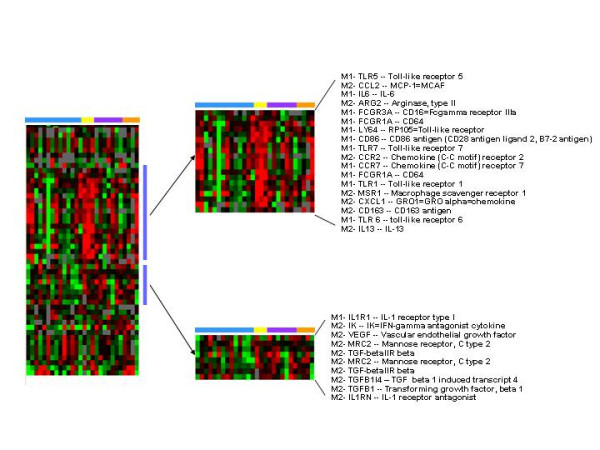
**Analysis of the expression of macrophage genes**. Genes characteristic of M1 and M2 macrophages were selected according to a list of transcripts reported in the literature [21,22,25] to be modulated by these cell subsets and were analyzed by supervised hierarchical clustering which revealed two clusters. The first cluster was a mix of M1 and M2 genes whose expression was greatest on day 2. The second cluster was predominantly M2 genes and their expression was greatest on day 4 and 8. Pre and post wound biopsies are color coded by the horizontal bars as per figure 1.

Several macrophage markers were expressed both early and late in wound healing. During many inflammatory processes macrophages have been found to be polarized as M1 or M2 macrophages [21-24]. To determine the polarization of the macrophage activation, all macrophage genes whose expression changed were selected, (see additional file 2) and supervised hierarchical clustering was used to analyze the expression of these M1 and M2 macrophage genes in the pre- and post-wound samples from all 14 subjects. This information was compared to reported assignment of genes to M1 or M2 according to the literature [21,22]. Two clusters of genes were identified. One cluster contained genes whose expression was increased early, on day 2, and the other genes whose expression was increased later, on days 4 and 8 (Figure 6). The early cluster was made up of 18 genes and included a mix of M1 and M2 markers; eleven M1 markers and seven M2 markers. In contrast, the late expressed genes were predominantly M2 markers with nine M2 markers and one M1 marker.

## Discussion

The wound healing process occurring in lesions of basal cell carcinoma was evaluated on days 2 through 8 after a cutaneous punch biopsy wound. We found that wound healing is more than a simple orderly progression of cells involved with acute inflammation and tissue repair. The classically described model of wound healing states that by day 2, the hemostasis phase of wound healing should be complete. Days 2 through 8 should include the classical inflammatory, proliferative, and repair phases. However, we found no distinct separation between inflammation, repair, remodeling, proliferation and angiogenesis. Instead, we found that acute wound healing was associated with a storm of transcripts involved in repair, remodeling and inflammation and angiogenesis which increased at all time points.

Nearly all genes whose expression changed were up regulated, and most of these upregulated genes were involved with inflammation. We identified 3 different groups of genes whose expression was increased. The largest group included those whose expression was increased throughout the study period and was made up predominantly of inflammatory transcripts. This group included markers of a wide variety of leukocytes including macrophages, granulocytes, T cells, NK cells, and B cells. Markers of tissue repair and angiogenesis were also expressed throughout the study, although to a much lesser extent than the inflammatory transcripts.

The second and third groups consisted of genes whose expression was transiently increased after wound infliction. The expression of an early group of markers was greatest on day 2 and the expression of the other group of markers was greatest on days 4 and 8. Both groups of genes included a mix of markers of inflammation, repair and angiogenesis, however, inflammation markers were predominant in the early group and repair and angiogenesis markers in the later group.

Classical models view wound healing as being directed largely by cytokines and growth factors [2]. Our study suggests that both the presence and phenotype of macrophages bears consideration in wound healing as a potentially guiding element in normal repair.

The expression of a number of macrophage genes were increased throughout the study. Macrophages are potent producers of cytokines and growth factors and are known to play important roles in autoimmunity, defense against infection, response to cancer, tissue repair, tissue remodeling and angiogenesis. Macrophages also have diverse phenotypes [21,22,25]. M1 polarized macrophages are important in inflammation. They are potent effector cells that produce large quantities of proinflammatory cytokines and kill microorganisms and cancer cells [22]. M2 macrophages tune the inflammatory responses, scavenge debris, and promote angiogenesis, tissue remodeling and repair [22]. We found that the profile of macrophage related transcripts changed from a mixture of M1 and M2 genes early in wound healing to primarily M2 genes later. Interestingly, Martinez and colleagues have found that monocytes in the presence of macrophage colony-stimulating factor (M-CSF), a growth factor found at high concentrations in blood from healthy subjects, drift forward a M2 phenotype suggesting that M2 may be a default pathway of macrophage differentiation [26]. Early in wound healing M1 macrophages likely direct an inflammatory response that helps clear the wound of microbes, cellular deébris, and damaged matrix and M2 macrophages initiate repair and angiogenesis. Late in wound healing, the dominant M2 macrophages continue to direct tissue repair and angiogenesis. One type of M2 macrophage, tumor associated macrophages, produce TGF-β and VEGF [21,22,25]. It may be that macrophages rather than TGF-β and VEGF drive the change in wound healing from a primary inflammatory process to one of repair, angiogenesis, and remodeling.

The conclusions that can be drawn from this study are somewhat limited since gene expression analysis was done on punch biopsies derived from skin with basal cell carcinoma. Previous studies have concluded that cancer cells are wounds that never heal [27] and that prior to the wound, skin with basal cell carcinoma would be expected to contain both inflammatory cells and cells involved with remodeling, repair, and angiogenesis. Against this background of inflammation, angiogenesis and remodeling that occurs with cancer, our study may not have been able to detect some gene expression changes associated with wound healing occurring in normal tissue. In fact, this background of healing and inflammation associated with the basal cell carcinoma may explain the inability of unsupervised clustering analysis to separate some pre- and post-wound samples. We compared changes in gene expression in the healing skin with a pre wound sample of skin with basal cell carcinoma rather than with normal skin. While the differences detected likely reflect changes due to healing rather than responses to the basal cell carcinoma, these observations should be confirmed in healing normal skin.

Despite the limitations of this study, our results expand the current understanding of wound healing by offering a snapshot of the concomitantly occurring transcriptional events that characterize the overlapping phases of this biological process. In addition, our results confirm previous reports describing the occurrence of several crucial factors involved wound healing which we have found highly upregulated at the mRNA level. We found that the expression of several genes previously noted to be involved with wound healing, angiogenesis and tissue repair were increased. These genes included S100A, progranulin, MMP-9, αv integrin, β5 integrin, metallothioneins, and connective tissue growth factor.

Early in wound healing S100A4 expression was increased. S100A4 belongs to the S100 family of calcium binding proteins. It is produced by macrophages, leukocytes, fibroblast and endothelial cells [28]. Extracellular S100A4 is involved with angiogenesis and the remodeling of extracellular matrix. It is involved with tumor progression and may contribute to synovium hyperplasia in rheumatoid arthritis [28].

An important wound healing growth factor progranulin was among the late expressed genes. Progranulin is also known as granulin or epithelin precursor, acrogranin, and PC-derived growth factor [4]. Progranulin is a pluripotential growth factor that promotes tissue granulation and neovascularization [4,29]. Progranulin activates extracellular kinases. It is expressed on many tissues throughout the body especially by epithelial cells undergoing rapid turnover. During wound healing progranulin mRNA levels are upregulated in the dermis for at least 10 days following the wound [29]. The administration of progranulin to wounds prolongs neutrophil infiltration and increases the accumulation of fibroblasts and blood vessels in the wound by promoting dermal fibroblast and endothelial cell proliferation and their migration to type I collagen [29].

MMP-9 was also among the late expressed genes. MMP-9 is a metalloproteinase which promotes angiogenesis indirectly by making VEGF more available to its receptor. MMP-9 has been shown to promote angiogenesis in cancers [30]. The enhanced expression of MMP-9 during days 4 through 8 of wound healing suggests that it may also contribute to angiogenesis during wound healing.

Other wound healing genes whose expression we found to be increased late were the integrins αv and β5. The integrin αv is important in angiogenesis, fibroblast migration and wound closure. αvβ5 is a receptor for vitronectin and αvβ3 is a receptor for both fibronectin and vitronectin. Both αvβ3 and αvβ5 play a major role in blood vessel formation in cancer, arthritis, and ischemic retinopathy [31]. Fibroblast migration to an angiogenic factor CYN61 is mediated by αvβ5. In addition during normal wound healing fibroblasts differentiate into myofibroblasts in granulation tissue where they play a role in wound contraction and scar formation [32]. The development of myofibroblasts is dependent on αvβ3 and αvβ5 [32].

Connective tissue growth factor (CTGF) or CCN2 [33,34] is an important wound healing growth factor that promotes fibrosis. CCN2 is not normally expressed in dermal fibroblasts, but during wound healing fibroblasts express CCN2. In this study the expression of CCN2 was increased on days 4 and 8. CCN2 promotes matrix deposition, fibrobast proliferation and fibroblast adhesion.

The expression of three methallothioneins, methallothionein 1G, 2A and G, were increased throughout the study [35]. Methallothioneins are a family of cysteine-rich low molecular weight proteins that bind trace metals including zinc and copper. The expression of methallothioneins is increased in metabolically active and proliferating cells of the dermis. In skin wounds the levels of methallothioneins are increased in the wound margin where mitotic activity is increased. Methallothioneins are thought to promote cell proliferation and re-epithelialization through its zinc and copper binding properties [35].

In conclusion, cutaneous wounds are initially associated with increased expression of genes involved with all aspects of inflammation and repair. Over the course of healing the profile of genes expressed changes from predominantly inflammation to one of repair and angiogenesis. This shift in gene profile may reflect at least in part the participation of M2 macrophages.

## Supplementary Material

Additional File 1 Differentially expressed inflammatory, immune response, and wound healing genes that were not among the signature clusters. Click here for file

Additional File 2 Genes related to M1 or M2 polarization that had increased expression post-wounding. Click here for file
