# Supplementary material for: Gene expression profiling of cutaneous wound healing
Source: J Transl Med. 2007 Feb 21;5:11. doi: 10.1186/1479-5876-5-11 (PMC1804259; doi:10.1186/1479-5876-5-11)
Supplement: Additional File 1 — Differentially expressed inflammatory, immune response, and wound healing genes that were not among the signature clusters. [file 1479-5876-5-11-S1.doc]

**Additional File 1.** **Differentially Expressed Inflammatory, Immune Response, and Wound Healing Genes that were not Among the Signature Clusters***

| **When Gene Expression was Increased** | **Image Number** | **Gene Name** |
| --- | --- | --- |
| Day 2 | 310148 | ACVRL1 -- TGF beta receptor type I |
|  | 2430220 | S100A4 -- S100 calcium binding protein A4 |
|  | 154185 | CD68 -- CD68 antigen |
|  | 290234 | ITGAX -- Integrin, alpha X (antigen CD11C (p150), alpha polypeptide) |
|  | 2417330 | CDW52 -- CDW52 antigen (CAMPATH-1 antigen) |
|  | P05293 | IL24 -- interleukin 24 |
|  | 484898 | EBI2 -- EBI2=Epstein-Barr virus induced G-protein coupled receptor |
|  | P04763 | CXCR4--chemokine (C-X-C motif), receptor 4 |
|  | 713145 | CD44 -- CD44=Pgp-1=extracellular matrix receptor-III |
|  | 840677 | Ig light chain gene variable domain (CLL-L3B) |
|  | 840677 | Ig light chain gene variable domain (CLL-L3B) |
| Days 4 and 8 | 294196 | LNK -- Lymphocyte adaptor protein |
|  | 2406134 | MAPKAPK3 -- Mitogen-activated protein kinase-activated protein kinase 3 |
|  | 132702 | P4HB -- Procollagen-proline, 2-oxoglutarate 4-dioxygenase |
|  | 43550 | LDHA -- Lactate dehydrogenase A |
|  | 2543381 | CD99 -- CD99 antigen |
|  | 1435862 | CD99 -- CD99 antigen |
|  | 840978 | CD81 -- CD81=TAPA1 |
|  | 198453 | HLA-E -- MHC Class I=HLA-E |
|  | 198453 | HLA-E -- MHC Class I=HLA-E |
|  | 1602516 | DAXX -- Death-associated protein 6 |
|  | 488019 | IL15RA -- IL-15 receptor alpha chain |
|  | 753278 | CXCL16 -- CXCL16=transmembrane CXC chemokine |
|  | 469969 | ITGAV -- Integrin, alpha V (antigen CD51) |
|  | 2450237 | SOCS3 -- SOCS-3 |
|  | 469969 | ITGAV -- Integrin, alpha V |
|  | 2450237 | SOCS3 -- SOCS-3=STAT induced STAT inhibitor-3 |
|  | 362278 | SOCS3 -- Suppressor of cytokine signaling 3 |
|  | 713782 | ADAM15 -- A disintegrin and metalloproteinase domain 15 (metargidin) |
|  | 127821 | ACP5 -- TRAP-5 (tartrate-resistant acid phosphatase) |
|  | 1759582 | TNFRSF12A -- Tumor necrosis factor receptor superfamily, member 12A |
|  | P08770 | platelet-derived growth factor receptor, beta polypeptide |
|  | 154173 | COL5A3 -- Collagen, type V, alpha 3 |
|  | 898092 | CTGF -- Connective tissue growth factor |
|  | 1474174 | MMP2 -- Matrix metalloproteinase 2 |
|  | 202498 | IL10RB -- IL-10 receptor beta |
|  | 669443 | HSF2 -- Heat shock transcription factor 2 |
|  | 727026 | ADAMTS3 -- A disintegrin-like and metalloprotease with thrombospondin |
| Days 2 through 8 | 2326019 | COX5B -- Cytochrome c oxidase subunit Vb |
|  | 2505796 | COX5B -- Cytochrome c oxidase subunit Vb |
|  | 2317017 | MT1G -- Metallothionein 1G |
|  | 812251 | MAPKAPK2 -- Mitogen-activated protein kinase-activated protein kinase 2 |
|  | 2306804 | PSMD13 -- Proteasome (prosome, macropain) 26S subunit, non-ATPase, 13 |
|  | 2549900 | PSMD13 -- Proteasome (prosome, macropain) 26S subunit, non-ATPase, 13 |
|  | 2568305 | BCAP31 -- B-cell receptor-associated protein 31 |
|  | 2561977 | TIA1 -- TIA1 cytotoxic granule-associated RNA binding protein |
|  | 2571344 | EDF1 -- Endothelial differentiation-related factor 1 |
|  | 2565981 | MIF -- Macrophage migration inhibitory factor |
|  | 1574438 | MMP11 -- Matrix metalloproteinase 11 |
|  | 510383 | IFITM1 -- Interferon-inducible protein 9-27 |
|  | 590150 | MT2A -- metallothionein 2A |
|  | 1592837 | IFITM2 -- Interferon induced transmembrane protein 2 (1-8D) |
|  | 809910 | IFITM3 -- Interferon-inducible protein 1-8U |
|  | 809910 | IFITM3 -- Interferon-inducible protein 1-8U |
|  | 1492238 | MAPBPIP -- Mitogen-activated protein-binding protein-interacting protein |
|  | 2508733 | LY75 -- Lymphocyte antigen 75 |
|  | 161993 | CEBPB -- NF-IL6=C/EBP beta |
|  | 784112 | COL5A2 -- Collagen, type V, alpha 2 |
|  | 182999 | DUSP22 -- Dual specificity phosphatase 22 |
|  | 298268 | BCL7B -- B-cell CLL/lymphoma 7B |
|  | 2580561 | HLA-B -- Major histocompatibility complex, class I, B |
|  | 810142 | HLA-C -- MHC Class I=HLA-C4 |
|  | 1613052 | CLSTN1 -- Calsyntenin 1 |
|  | 811813 | HSPA1B -- Heat shock 70kDa protein 1B |
|  | 188232 | KLF4 -- GKLF=EZF=KLF4=gut-enriched Kruppel-like zinc finger protein |
|  | 2019011 | MT3 -- Metallothionein 3 (growth inhibitory factor (neurotrophic)) |
|  | 298268 | BTG1 -- BTG1=B-cell translocation gene 1=anti-proliferative |
|  | 1455976 | IFITM2 -- Interferon induced transmembrane protein 2 (1-8D) |
|  | 809910 | IFITM3 -- Interferon-inducible protein 1-8U |
|  | 2572015 | TIMP1 -- Tissue inhibitor of metalloproteinase 1 |
|  | 755599 | IFITM1 -- Interferon induced transmembrane protein 1 (9-27) |
|  | 1358393 | MAP2K3 -- Mitogen-activated protein kinase kinase 3 |
|  | 1560599 | TGF-betaIIR beta |
|  | 26568 | EGR3 -- EGR-3=PILOT=T cell transcription factor=bridging integrator 3 |
|  | 2565853 | COX5A -- Cytochrome c oxidase subunit Va |
|  | 120881 | RAB31 -- RAB31, member RAS oncogene family |
|  | 344589 | LCP1 -- L-plastin=actin-binding protein |
|  | 130541 | PECAM1 -- Platelet/endothelial cell adhesion molecule (CD31 antigen) |
|  | 447509 | HLA-DOA -- MHC Class II=DN alpha |
|  | 840511 | VIM -- Vimentin |
|  | 840691 | STAT1 -- STAT1 |
|  | 1535851 | COX15 -- COX15 homolog, cytochrome c oxidase assembly protein (yeast) |
|  | 711993 | IFNGR1 -- interferon-gamma receptor alpha chain |
|  | 27548 | NUP153 -- Nucleoporin 153kDa |
|  | 714106 | PLAU -- Plasminogen activator, urokinase |
|  | 122241 | PSMB2 -- Proteasome (prosome, macropain) subunit, beta type, 2 |
|  | 325117 | HIF1A -- HIF-1 alpha=hypoxia-inducible factor 1 alpha |
|  | 1536452 | RAB14 -- RAB14, member RAS oncogene family |
|  | 32493 | ITGA6 -- CD49F=Integrin alpha 6 |
|  | 487773 | ADAM10 -- A disintegrin and metalloproteinase domain 10 |
|  | 33826 | MAP2K1 -- mitogen-activated protein kinase kinase 1 |
|  | 2316471 | DEDD -- Death effector domain containing |
|  | 2504881 | STAT5A -- STAT5A |
|  | 241481 | CASP10 -- CASPASE-10 |
|  | P22597 | TIMP1=tissue inhibitor of metalloproteinase 1 |
|  | P08770 | ICSBPI -- interferon consensus sequence binding protein 1 |
|  | 2009477 | CD6 -- CD6 antigen |
|  | 1928791 | F3 -- Coagulation factor III (thromboplastin, tissue factor) |
|  | 714453 | IL4R -- IL-4 receptor alpha chain |
|  | 809707 | JUNB -- jun-B |
|  | 745143 | CASP6 -- Caspase 6, apoptosis-related cysteine protease |
|  | 2541366 | AQP3 -- Aquaporin 3 |
|  | 2291894 | EVER1 -- LAK-4p=enhanced expression with T/LAK-cell-activation |
|  | 2466502 | PSMC4 -- Proteasome (prosome, macropain) 26S subunit, ATPase, 4 |
|  | 156343 | MAP3K3 -- Mitogen-activated protein kinase kinase kinase 3 |
|  | 2314294 | FCER1A -- Fc fragment of IgE, high affinity I, receptor for; alpha polypeptide |
|  | 1631863 | HLA-DQB2 -- Major histocompatibility complex, class II, DQ beta 2 |
|  | 491460 | LTBP4 -- Latent transforming growth factor beta binding protein 4 |
|  | 37234 | MAP4K2 -- mitogen-activating protein kinase kinase kinase kinase 2 |
|  | 810331 | QSCN6 -- BPGF-1=bone-derived growth factor=Q6 |
|  | 809946 | IFRD2 -- Interferon-related developmental regulator 2 |
|  | 1572298 | CD3Z -- CD3Z antigen, zeta polypeptide (TiT3 complex) |
|  | 704532 | NMI -Nmi=IL-2 and IFN-gamma inducible potentiator of STAT -induced transcription |
|  | 504527 | DUSP1 -- dual specificity phosphatase 1=MKP-1=CL100 |
|  | 309864 | JUNB -- Jun B proto-oncogene |
|  | 460487 | LTF -- lactotransferrin |
|  | 1947606 | TLR1 -- Toll-like receptor 1 |
|  | 1901363 | KLRD1 -- Killer cell lectin-like receptor subfamily D, member 1 |
|  | P33111 | Granulocyte-macrophage colony stimulating factor 2 receptor |
|  | 811024 | BST2 -- bone marrow stromal cell antigen 2 |
|  | 742132 | G1P2 -- Interferon-induced 17 KD protein |
|  | 823696 | IFIT1 -- Interferon-induced 56-KDa protein |
|  | 782513 | G1P3 -- Interferon, alpha-inducible protein (clone IFI-6-16) |
|  | P04592 | interferon-induced, hepatitis C-associated microtubular aggregate protein |
|  | 1456118 | PSMB9 -- Proteasome (prosome, macropain) subunit, beta type, 9 |
|  | 878798 | B2M -- Beta-2-microglobulin |
|  | 1534435 | TIMP2 -- Tissue inhibitor of metalloproteinase 2 |
|  | 2566009 | TIMP2 -- Tissue inhibitor of metalloproteinase 2 |
|  | 1604703 | HLA-F -- Major histocompatibility complex, class I, F |
|  | P48604 | interferon gamma receptor 2 (interferon gamma transducer 1) |
|  | 376475 | TNFSF13 -- Tumor necrosis factor (ligand) superfamily, member 12 |
|  | 813756 | AP1S2 -- Adaptor-related protein complex 1, sigma 2 subunit |
|  | 299559 | RAB35 -- RAB35, member RAS oncogene family |

*These genes were differentially expressed during wound healing, but were not part of the original signature genes (Figure. 1). The results of clustering of these genes is shown in figure 5.
