# Supplementary material for: Gene expression profiling of cutaneous wound healing
Source: J Transl Med. 2007 Feb 21;5:11. doi: 10.1186/1479-5876-5-11 (PMC1804259; doi:10.1186/1479-5876-5-11)
Supplement: Additional File 2 — Genes related to M1 or M2 polarization that had increased expression post-wounding. [file 1479-5876-5-11-S2.doc]

**Additional file 2. Genes Related to M1 or M2 Polarization that had Increased Expression Post-Wounding**

|  | **Image ID** | **Gene Name** |
| --- | --- | --- |
| M1 Macrophage  Genes | 503617 | CXCL9 -- Mig=Humig=chemokine targeting T cells |
|  | 1493160 | CXCL10 -- Chemokine (C-X-C motif) ligand 10 |
|  | 2345206 | CCR7 -- Chemokine (C-C motif) receptor 7 |
|  | 80633 | TLR2 -- Toll-like receptor 2 |
|  | 810213 | IL1R1 -- IL-1 receptor type I |
|  | 795343 | FCGR1A -- CD64=high affinity immunogobulin gamma FC receptor I |
|  | 795343 | FCGR1A -- CD64=high affinity immunogobulin gamma FC receptor I |
|  | 868380 | FCGR2A -- Fc fragment of IgG, low affinity IIa, receptor for (CD32) \|\| |
|  | 51447 | FCGR3A -- CD16=Fcgamma receptor IIIa \|\| |
|  | 1569006 | CD86 -- CD86 antigen (CD28 antigen ligand 2, B7-2 antigen) |
|  | 80633 | TLR2 -- Toll-like receptor 2 |
|  | EnaW-9k-97h2 | TLR 6 -- toll-like receptor 6 |
|  | 826220 | LY64 - RP105=Toll-like receptor that cooperates with TLR4 |
|  | 257746 | TLR7 -- Toll-like receptor 7 |
|  | 1947606 | TLR1 -- Toll-like receptor 1 |
|  | 144675 | TLR3 -- Toll-like receptor 3 |
|  | 277229 | TLR5 -- Toll-like receptor 5 |
|  | 1579639 | IFNG -- Interferon gamma |
|  | 310406 | IL6 -- IL-6 |
| M2 Macrophage Genes | 768497 | CCL18 -- PARC=DC-CK1=CC chemokine targeting T cells, not monocytes |
|  | 1475375 | SRCRB4D -- Scavenger receptor cysteine rich domain containing, group B ( |
|  | 841220 | scavenger receptor class B, member 1 |
|  | 1592058 | MRC2 -- Mannose receptor, C type 2 |
|  | 48886 | MRC1 -- mannose receptor, C type 1 |
|  | 204539 | CCR2 -- Chemokine (C-C motif) receptor 2 |
|  | 768561 | CCL2 -- MCP-1=MCAF=small inducible cytokine A2=JE=chemokine |
|  | 153340 | CXCL2 -- Chemokine (C-X-C motif) ligand 2 |
|  | 324437 | CXCL1 -- GRO1=GRO alpha=melanoma growth stimulatory activity |
|  | 45138 | VEGFC -- vascular endothelial growth factor related protein VRP |
|  | 855061 | VEGFB -- Vascular endothelial growth factor B |
|  | 34778 | VEGF -- Vascular endothelial growth factor |
|  | 1560599 | TGF-betaIIR beta |
|  | 136821 | TGFB1 -- Transforming growth factor, beta 1 (Camurati-Engelmann disease) |
|  | 868630 | TGFB1I4 -- Transforming growth factor beta 1 induced transcript 4 |
|  | 609087 | SCARB2 -- Scavenger receptor class B, member 2 |
|  | 841220 | scavenger receptor class B, member 1 |
|  | 756687 | SCARB1 -- Scavenger receptor class B, member 1 |
|  | 1571913 | TU3A -- TU3A protein |
|  | 813635 | ARG1 -- Arginase, liver |
|  | 204539 | CCR2 -- Chemokine (C-C motif) receptor 2 |
|  | 727292 | CD163 -- CD163 antigen |
|  | 84295 | IL1RN -- IL-1 receptor antagonist |
|  | 858911 | SCARB2 -- Scavenger receptor class B, member 2 |
|  | 1946722 | MSR1 -- Macrophage scavenger receptor 1 |
|  | 1837472 | IL13 -- IL-13 |
|  | 23282 | IK -- IK=IFN-gamma antagonist cytokine |
|  | 1874367 | CCL20 -- Chemokine (C-C motif) ligand 20 |
|  | 2150502 | CCL22 -- MDC=STCP-1=macrophage-derived chemokine precursor |
|  | 50480 | ARG2 -- Arginase, type II |
